# The role of Lutheran/basal cell adhesion molecule in human bladder carcinogenesis

**DOI:** 10.1186/s12929-017-0360-x

**Published:** 2017-08-26

**Authors:** Hong-Yi Chang, Hsin-Mei Chang, Tsung-Jung Wu, Chang-Yao Chaing, Tzong-Shin Tzai, Hong-Lin Cheng, Giri Raghavaraju, Nan-Haw Chow, Hsiao-Sheng Liu

**Affiliations:** 1 Institute of Basic Medical Sciences, College of Medicine, National Cheng Kung University, Tainan, Taiwan, Republic of China; 2 Department of Urology, College of Medicine, National Cheng Kung University, Tainan, Taiwan, Republic of China; 3 Department of Microbiology and Immunology, College of Medicine, National Cheng Kung University, Tainan, Taiwan, Republic of China; 4 Department of Pathology, College of Medicine, National Cheng Kung University, Tainan, Taiwan, Republic of China; 5 Center of Infectious Disease and Signaling Research, College of Medicine, National Cheng Kung University, Tainan, Taiwan, Republic of China

**Keywords:** Lutheran, Basal cell adhesion molecule, Laminin, Urothelial cancer, Ras

## Abstract

**Background:**

Lutheran/basal cell adhesion molecule (Lu/BCAM) is a membrane bound glycoprotein. This study was performed to investigate the role and downstream signaling pathway of Lu/BCAM in human bladder tumorigenesis.

**Methods:**

Five human bladder cancer (E6, RT4, TSGH8301, TCCSUP and J82), one stable mouse fibroblast cell line (NIH-Lu) expressing Lu/BCAM transgene and sixty human uroepithelial carcinoma specimens were analyzed by real-time PCR, immunohistochemistry (IHC), immunofluorescence (IFA) staining, Western blotting and promoter luciferase assay for *Lu/BCAM*, respectively. The tumorigenicity of Lu/BCAM was demonstrated by focus formation, colony-forming ability, tumour formation, cell adhesion and migration.

**Results:**

H-*ras* ^*V12*^ was revealed to up-regulate *Lu/BCAM* at both transcriptional and translation levels. Lu/BCAM expression was detected on the membrane of primary human bladder cancer cells. Over-expression of Lu/BCAM in NIH-Lu stable cells increased focus number, colony formation and cell adhesion accompanied with F-actin rearrangement and decreased cell migration compared with parental NIH3T3 fibroblasts. In the presence of laminin ligand, Lu/BCAM overexpression further suppressed cell migration accompanied with increased cell adhesion. We further revealed that laminin-Lu/BCAM-induced cell adhesion and F-actin rearrangement were through increased Erk phosphorylation with an increase of RhoA and a decrease of Rac1 activity. Similarly, high Lu/BCAM expression was detected in the tumors of human renal pelvis, ureter and bladder, and was significantly associated with advanced tumor stage (*p* = 0.02). Patients with high Lu/BCAM expression showed a trend toward larger tumor size (*p* = 0.07) and lower disease-specific survival (*p* = 0.08), although not reaching statistical significance.

**Conclusion:**

This is the first report showing that Lu/BCAM, in the presence of its ligand laminin, is oncogenic in human urothelial cancers and may have potential as a novel therapeutic target.

**Electronic supplementary material:**

The online version of this article (doi:10.1186/s12929-017-0360-x) contains supplementary material, which is available to authorized users.

## Background

Tumor cells invasion/metastasis from basement membrane is a critical stage in tumor progression [1]. During metastasis, tumor cells acquire multiple biological properties, including overcoming contact inhibition, remodeling cytoskeleton and modulating cell adhesion [2]. Accumulating evidence indicates that alteration of cell adhesion plays a pivotal role in cancer metastasis because of activation of diverse intracellular signaling molecules such as Ras, Erk and small Rho-GTPases [3, 4]. The interaction between cancer cells and extracellular matrix (ECM) promotes carcinogenic processes including cell growth, survival, migration, extravasation, homing and metastasis [5]. As a result, adhesion molecules have been proposed as the potential therapeutic targets [6].

Lu/BCAM (Lutheran/basal cell-adhesion molecule, a glycoprotein) belongs to the immunoglobulin superfamily which contains both Lu blood group and BCAM tumor-associated antigens. This transmembrane Lu/BCAM protein consists of two isoforms: Lu (628 amino acid) and Lu (v13) (588 amino acid), which differ in the size of cytosolic domain. Lu/BCAM contains the binding sites for SH3 domain and phosphorylation for signal transduction. It is known that Lu/BCAM functions as the receptor for specific ligand laminin-α5 chain, a major component of the extracellular matrix proteins, such as laminin-10 (α5β1γ1), −11 (α5β2γ1) and −15 (α5β2γ3) [7–9].

The significance of Lu/BCAM on tumorigenesis has been increasingly recognized. Lu/BCAM is involved in cell-cell adhesion and migration in skin tumor [10, 11]. Maatta et al. reported that non-polarized expression of Lu/BCAM in epithelial ovarian cancer participates in local progression [12]. Furthermore, co-expression of Lu/BCAM and integrin on the cell surface is responsible for adhesion of hepatocellular carcinoma cells [9]. Proteomics analysis suggests that BCAM might be a potential biomarker for pancreatic cancer [13]. Therefore, Lu/BCAM appears to plays an oncogenic role during tumorigenesis, however, the underlying molecular mechanisms and downstream signaling pathways of Lu/BCAM in tumorigenesis remain elusive.

In this study, we compared the gene expression profile between a primary human bladder epithelial cell line – E6 and its derivative E6RC which harbors an isopropyl-β-D-thiogalactopyranoside (IPTG, an analogy of lactose) inducible H-*ras* ^V12^ oncogene using a cDNA microarray screen platform. Lu/BCAM is one of the ras oncoprotein up-regulated genes on this basis, we intended to clarify the role of Lu/BCAM during tumorigenesis of human bladder. Firstly, the mouse fibroblast NIH3T3 cell and its derivative were utilized because it is widely used to characterize unknown oncogenes [14]. Then, human bladder cancer cell lines was chosen to clarify the tumorigenicity of Lu/BCAM and its the underlying mechanisms in the presence or absence of laminin-10/11 ligand.

## Methods

### Cell culture, stable cells, chemicals, antibodies and plasmids

Mouse NIH3T3 fibroblasts were obtained from the American Type Culture Collection (ATCC). Human embryonic kidney cell (HEK293; ATCC) and E6 immortalized human uroepithelium [15] were maintained in Dulbecco’s Modified Eagle’s Medium (DMEM; GIBCO) supplemented with 10% Fetal bovine serum (GIBCO) at 37 °C in a 5% CO_2_ incubator. Human bladder cancer cell lines (RT4, TCCSUP and J82) [16] were also purchased from ATCC. These cells were maintained in DMEM as previously described [17, 18]. TSGH8301 cell was from the Bioresource Collection and Research Center (BCRC; Taiwan). To establish the stable cell line expressing the exogenous Lu/BCAM gene, The plasmid pcDNA3.1-Lu was transfected into NIH3T3 cells by calcium phosphate transfection followed by G418 (300 μg/ml) selection. Two stable cell lines NIH-Lu10 and NIH-Lu11 were established, and the expression of Lu was confirmed by real-time PCR. Ras antibody was purchased from Calbiochem, USA, and the antibodies to phosphorylated Erk, p38 MAPK and JNK were obtained from Cell Signaling, USA. The antibody to RhoA was obtained from Santa Cruz Biotechnology, USA, and the antibody to Rac1 was obtained from BD Transduction laboratory, USA. Erk inhibitor PD98059 was obtained from Calbiochem. Wortmannin, C3 and human placenta laminin-10/11 (laminin), the ligand of Lu/BCAM [19, 20], were purchased from Sigma. The plasmid pcDNA3.1-Lu was provided by Dr. Marilyn J. Telen (Duke University Medical Center, NC, USA). The plasmids of pGST-C21, pGST-PAK, pGST were provided by Dr. Hong-Chen Chen (National Chung Hsing University, Taichung, Taiwan).

### Cell lysis and western blotting

Cell lysate was prepared as previously described [21]. Briefly, cells were washed twice with ice-cold PBS and lysed with 200 μl of whole-cell extract lysis buffer per 10-cm plate (50 mM Tris, pH 7.4, 1% NP40, 2 mM EDTA, 100 mM NaCl, 10 mM Na_3_VO_4_, 0.1% SDS, 10 mg/ml leupeptin, 2 mg/ml aprotinin, and 100 mM phenylmethylsulfonyl fluoride) (protease inhibitors were from Roche Applied Sciences. Cell lysates were further cleaned by centrifugation at 14000 rpm for 15 min. Protein concentration was determined using a Bradford assay (Bio-Rad). For Western blot analysis, equal amounts of cell lysates were boiled for 5 min with the sample buffer before separation on a SDS-polyacrylamide gel [21]. The proteins in the gel were transferred onto a PVDF membrane (Millipore) in Tris-glycine buffer at 100 V for 1.5 h using an electroblotter (Amersham). Membranes were blocked with PBS containing 5% non-fat milk before incubating with primary antibodies. The protein complex was detected by enhanced chemiluminescence according to the manufacturer’s instructions (Millipore).

### RNA extraction, dual-luciferase reporter assay and reverse transcription-polymerase chain reaction (RT-PCR)

Total RNA was extracted using a single-step method with TRIzol™ reagent (Invitrogen). For RT-PCR, first strand cDNA was synthesized from 1 μg of total RNA with an oligo-dT primer and the Moloney murine leukemia virus (MMLV) reverse transcriptase (Promega). For dual-luciferase reporter assay, a 982 bp Lu/BCAM promoter fragment was amplified from genomic DNA by PCR using *Pfu* DNA polymerase and was cloned into the pGL3-basic promoter-less vector to generate the Lu-Luc reporter plasmid pGL3-Lupro. The luciferase reporter assay was performed as described previously [21].

### Cell transfection, RNA interference and real-time PCR

Cells in a six-well plate (2 × 10^5^/well) were transfected with 4 μg of pshRNA-Ras targeting different regions, psh-Ras-1 and psh-Ras-2 (Institute of Molecular Biology, Academia Sinica, Taipei, Taiwan), by Lipofectamine 2000™ following the manufacturer’s instructions (Invitrogen). The control vector was used pLKO.1. For real-time PCR, a Roche LightCycler™ real-time PCR system was used to measure the expression level of Lu/BCAM using SYBR Green I (Roche Applied Sciences) as the fluorescent dye. The following primers were used: Lutheran sense primer 5′- ctggaatggttccttaccg- 3′ and antisense 5′- caccacgcacacgtagtc- 3′. The primers of PPIA sense 5′-gtttgcagacaaggtccca −3′ and antisense 5′-acccgtatgctttaggatg- 3′ were used as an internal control. The real-time PCR was performed as described previously [21].

### Immunofluorescent staining and immunohistochemistry staining (IHC)

The cells seeded on the cover slide (2 × 10^5^) were fixed with 3.7% formaldehyde for 10 min and washed twice with PBS. The cells were then permeated with 0.1% Triton X-100 for 10 min. After blocking with 1% Bovine Serum Albumin (BSA) in PBS for 30 min, the cells were incubated with AlexaFluor™ 488-conjugated phalloidin (Molecular Probes Inc), which was used to stain F-actin or using M2-Flag monoclonal antibody (Sigma) to stain Flag fused Lu/BCAM under the fluorescence microscopy (Olympus). The IHC staining procedures were performed as described previously [22]. Briefly, tissue sections were incubated at RT for 2 h with anti-Lu antibody [22]. Then StrAviGen Super Sensitive MultiLink kit (BioGenex) was used to detect the resulting immune complex. Peroxidase activity was visualized using an amino ethyl carbazole substrate kit (Zymed). Because there was no apparent difference in staining intensity, only a proportion of tumor cells stained for Lu/BCAM was considered in the classification [23]. High level of Lu/BCAM expression means >50% of the tumor cells were positive by immune-staining. Low level of Lu/BCAM expression means 10%–50% of the tumor cells positively stained; and “negative” means <10% of the tumor cells were positively stained for Lu/BCAM protein.

### Soft agar and foci formation assay

Both NIH3T3 and NIH-Lu11 cells (1 × 10^4^) were mixed with 900 μl of 0.37% agar dissolved in DMEM containing 10% calf serum (GIBCO) in the presence or absence of laminin. After gently mixing, the mixture was layered over 1 ml of 0.6% basal agar in DMEM plus 10% calf serum in 6 well plates. Plates containing transformed cells form colonies within 14 days. Colonies with diameter larger than 3 μm were counted as previously described [24]. For the foci formation assay, cells were seeded on a 10-cm dish (1 × 10^3^/plate) containing DMEM. Cultures were fixed with 4% paraformaldehyde, stained with Giemsa and evaluated for foci formation after 14 days [23]. Foci formation was confirmed under a light microscope. Only colonies with the diameter greater than 3 μm were counted.

### Wound healing and cell adhesion assay

Cells (3 × 10^5^) were seeded on a 3-cm dish and cultured overnight. A midline wound was made on the monolayer cells and the wound healing process was recorded every 20 min until the wound was completely healed. The Image-Pro plus computer program (Media Cybernetics) was used to calculate the distance between wounded edges [15]. For cell adhesion, cells (4 × 10^3^/well) were incubated in the 96-well ELISA plates coated with laminin (10 μg/ml) or BSA (10 μg/ml) under serum starvation conditions for 60 min. Cells were then washed with PBS followed by fixation with 1% paraformaldehyde and staining with 0.1% crystal violet. The optical density was read at the wavelength of 595 nm in a microplate reader (Dynex).

### GST pull-down assay

*Escherichia coli* BL21 (DE3) harboring glutathione S transferase (GST)-PAK, GST-C21 or GST plasmid construct was grown at 37 °C to the mid-log phase of growth (A_600_ = 0.6). Expression of recombinant protein was induced by 0.1 mM of IPTG for 2 h at 37 °C. Cells were harvested, re-suspended in lysis buffer I (50 mM Tris HCl, pH 8.0; 50 mM EDTA and 2% Triton X-100) and then cell wall was broken by sonication. Cell lysates were incubated with glutathione agarose beads for 1 h at 4 °C. The protein-bound beads were washed three times with lysis buffer. Cells (4 × 10^6^) were harvested using lysis buffer II [20 mM Tris HCl (pH 8.0), 1% NP40, 137 mM NaCl, 1.5 mM MgCl_2_6H_2_O, 10% glycerol and 100 mM NaF]. Cell lysates were incubated for 1 h at 4 °C with GST-PAK or -C21 protein pre-coupled to glutathione-agarose beads to precipitate GTP-bound Rac and Rho, respectively. The precipitated complex was washed three times with lysis buffer and boiled in the sample buffer. Total lysates and precipitates were analyzed by immunoblotting.

### Human bladder cancer specimens and animal experiments

A total of 60 cases of frozen urothelial carcinoma specimens of the urinary tract were obtained from the archive (Table 1). Informed consents in accordance with the principles of the Declaration of Helsinki were signed by the patients with approval by the Institutional Review Board, National Cheng Kung University Hospital, Tainan, Taiwan (No.HR-97-117 and B-ER-102-021). The six-week-old female NOD/SCID mice were purchased from the Laboratory Animal Center of National Cheng Kung University, Tainan, Taiwan. The animals were maintained in a pathogen-free animal care facility. The experimental protocol complied with Taiwan’s Animal Protection Act and was approved by the Laboratory Animal Care and Use Committee of the National Cheng Kung University. These mice were inoculated subcutaneously with 1 × 10^6^ cells in 100 μl of PBS. Tumor volume was measured according to the formula V = 0.52 × a^2^ × b (a: smallest superficial diameter; b: largest superficial diameter).

**Table 1:** Correlation of Lutheran expression and biological indicators of human urinary tract cancer

Indicators	Positive		Negative	*P* value
	Low (+)	High (++)^b^		
Histological grade				0.12
Grade I	1	0	0	
Grade II	6	4	19	
Grade III	14	1	15	
Tumor shape				0.26
Papillary	10	4	14	
Non-papillary	10	1	20	
NA^a^	1	0	0	
Tumor size				0.07
≤ 1 cm	0	1	2	
> 1 to ≤ 3 cm	6	0	15	
> 3 to ≤ 5 cm	7	4	9	
> 5 cm	7	0	7	
N/A	1	0	1	
Multiplicity				0.75
Single	13	4	22	
Multiple	8	1	12	
Stage(TNM staging system)				.02#
pTa	2	3	8	
pT1	10	1	8	
pT2	1	0	10	
pT3–4	3	1	2	
pN+	2	0	6	
M+	3	0	0	

^a^ *N/A* not applicable; # *p* < 0.05

^b^ High level of expression indicates >50% of the tumor cells were immunostained. Low level of expression indicates 10%–50% reactivity; and “negative” indicates <10% staining for Lu protein. A total of 60 cases were analyzed

### Statistical analysis

The association between tumor staging or gross characteristics with expression status of Lu was analyzed by Chi-square test. The correlation between Lu/BCAM expression levels and disease-specific survival of cancer patients was constructed according to Kaplan-Meier method by Log rank test [25].

## Results

### Lu/BCAM was up-regulated by increasing *H-Ras*^*V12*^ expression in human cancer cells

Ras proteins may induce diverse effectors to modulate tumorigenesis-related gene expression and biological activities. To reveal the gene expression profile of H-*ras* ^*V12*^ oncoprotein in human urothelial cells, a c-DNA microarray screening was conducted on immortalized bladder uroepithelial cell line E6 and its derivative E6RC harboring the inducible *H-ras* ^*V12*^ oncogene in the presence of IPTG. The results showed that Lu/BCAM is one of the most up-regulated genes by H-*ras* ^*V12*^ in human uroepithelial cells (2.62 fold, Additional file 1: Table S1).

**Fig. 1:**
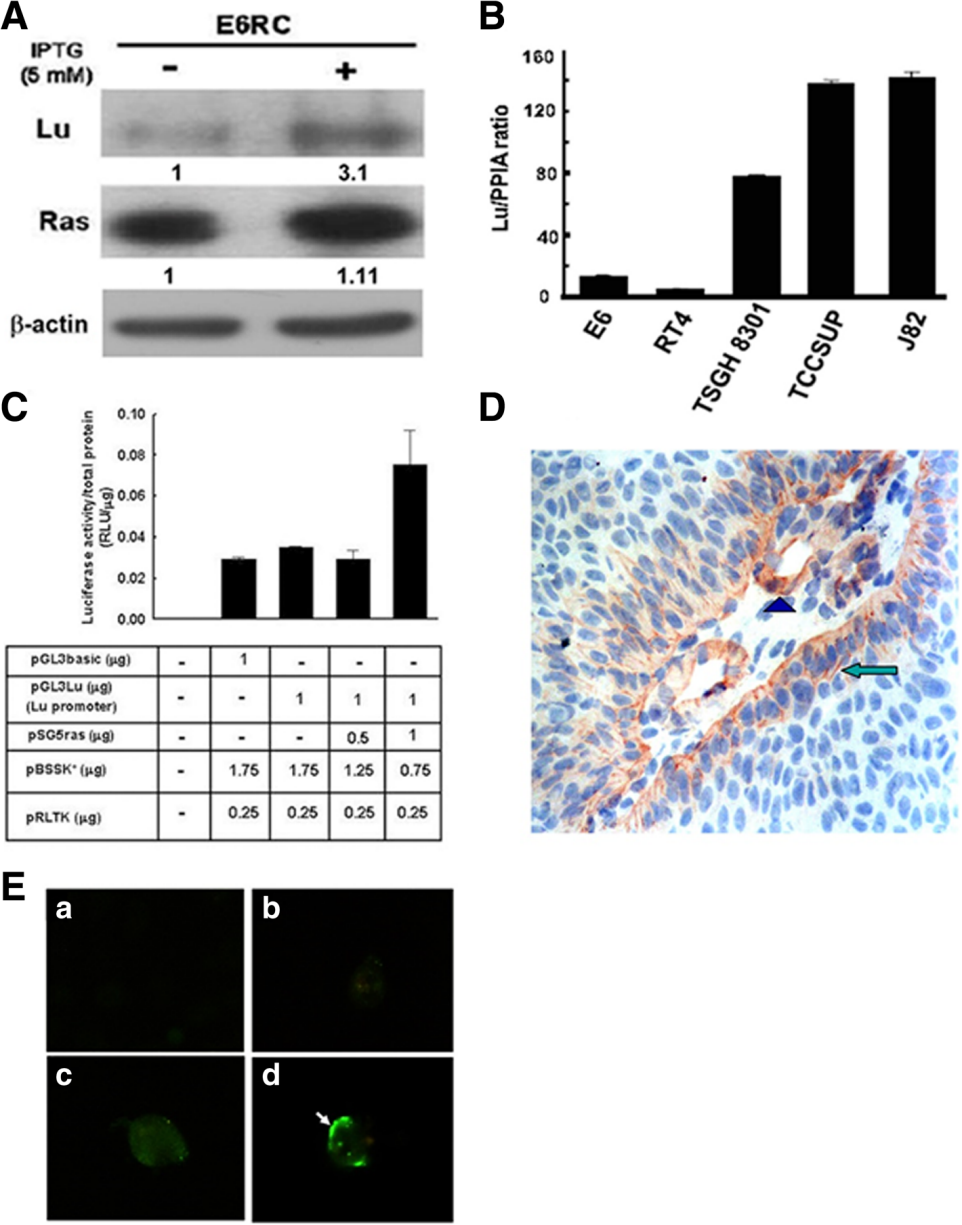
The relationship between H-*ras* ^*v12*^ and Lu/BCAM. (**A**) Expression levels of Lu/BCAM and Ras in human bladder uroepithelial cell line - E6RC harboring an inducible H-*ras* ^*V12*^ oncogene in the presence or absence of IPTG (5 mM) for 48 h were measured by immunoblotting. β-actin was used as an equal loading control. (**B**) The mRNA expression level of Lu/BCAM in five bladder cancer cell lines, i.e. immortalized (E6), early- (RT4), middle- (TSGH8301) and late-stage (TCCSUP and J82), was evaluated by real-time PCR. PPIA (peptidyl prolylisomerase A) was used as internal control to normalize the expression of Lu/BCAM. (**C**) The pGL3-Lu reporter plasmid together with pSG5*ras* (expressing H-*ras* ^*V12*^), pGL3basic and pBSSK^+^ were co-transfected into HEK293 cells and luciferase activity was determined using Dual-Luciferase™ Reporter assay system. The pRL-TK vector was used as an internal control for normalization. (**D**) Localization of Lu/BCAM protein in bladder cancer specimen by IHC staining using monoclonal anti-Lu antibody. Membranous expression of Lu/BCAM in the basal compartment of bladder carcinoma is indicated by *arrow*, *while* Lu expression in the endothelium and smooth muscle cells is shown by *arrowhead* (magnification: 300X; Olympus). (**E**) Localization of Lu/BCAM protein in TSGH8301 cells was detected by M2-flag monoclonal antibody using immunofluorescent assay under fluorescence microscopy (magnification: 400X; Olympus). **a**: Cell only; **b**: Cells transfected with vector plasmid pTRE2Hyg; **c**: Cells were co-transfected with plasmids of pTRE2Flag-Lu and pTet-Lac-Hyg, and then treated with doxycycline (1μg/ml); **d**: Cells received the same treatment as “**c**” except without doxycycline. *Arrow* points Lu protein

To validate the correlation between Ras and Lu/BCAM expression, we examined the protein expression levels in the E6RC stable cell line in the presence or absence of inducer IPTG. The Western blotting showed that Lu/BCAM expression was up-regulated when H-*ras* ^*Vl2*^ transgene expression was increased (Fig. 1A), which is consistent with the result of the microarray. To clarify the significance of Lu/BCAM expression in bladder cancer, five human uroepithelial cell lines were analyzed by real-time PCR. We showed that lower level of expression of Lu/BCAM was detected in E6 (primary immortalized) and RT4 (grade 1) cells, and higher level of Lu/BCAM was demonstrated in TSGH8301 (grade II), TCCSUP and J82 (grade III) cells (Fig. 1B). It implies that Lu/BCAM is positively associated with human bladder tumorigenesis.

To clarify whether H-*ras* ^*Vl2*^ regulates Lu/BCAM at the transcriptional level, Lu/BCAM promoter (pGL3-Lupro) and active form of H-*ras* ^*Vl2*^ (pSG5*ras*) plasmids were transiently co-transfected into HEK293 cells and luciferase activity of Lu/BCAM promoter was measured. An increase of Lu/BCAM promoter activity (2.6 fold) was up-regulated by H-*ras* ^*Vl2*^ at a higher dosage (Fig. 1C). Both Fig. 1A and C suggest that H-*ras* ^*Vl2*^ up-regulates Lu/BCAM expression at transcriptional and translational levels. Similar phenomenon was also observed in the stable human breast cancer cell line MCF-7-H-*ras* ^*V12*^ (Additional file 1: Fig. S1A). To further verify that expression of Lu/BCAM is regulated by H-*ras* ^*Vl2*^ protein, two *ras* specific small interfering RNAs (psh-Ras-1 and psh-Ras-2) were used to suppress H-*ras* ^*Vl2*^ protein expression. Accordingly, Lu/BCAM expression was decreased compared to negative (NC) and vector (V) control groups (Additional file 1: Fig. S1B), supporting that Lu/BCAM indeed is upregulated by H-*ras* ^*Vl2*^ protein.

### Lu/BCAM expression in human bladder cancer specimens and localization of Lu/BCAM protein in the bladder cancer cells

We then clarified the clinical significance of Lu/BCAM expression in sixty human urinary tract  cancer specimens by IHC staining. Our results showed that Lu/BCAM expression level is significantly correlated with tumor stage (*p* = 0.02). Lu/BCAM expression showed no significant difference in terms of  tumor grade, shape and multiplicity because of limited specimens (Table 1). It did show a trend toward correlation with tumor size (*p* = 0.07) and lower disease-specific survival (*p* = 0.08) (Additional file 1: Fig. S2). We also evaluated the adjacent non-tumor region. Expression of Lu/BCAM in the non-tumor uroepithelium was faint and the staining was close to background compared to tumor cells (data not shown). Nevertheless, analysis of more specimens is required to determine whether Lu is a marker of poor prognosis.

Furthermore, we examined the localization of Lu/BCAM in bladder cancer specimens and cancer cells in vitro. Membranous expression of Lu/BCAM was observed in the basal compartment of the primary bladder cancer (Fig. 1D, arrow) as well as in the endothelium and smooth muscle cells of lamina propria (Fig. 1D, arrowhead). The Lu/BCAM transgene expression was clearly located around the membrane of TSGH8301 bladder cancers cells by M2-Flag antibody staining (Fig. 1E-d) compared with vector control (Fig. 1E-b and c). Above results consistently showed that Lu/BCAM protein locates around cell membrane.

### The effect of Lu/BCAM overexpression on focus and colony formation in NIH-Lu11 cells

**Fig. 2:**
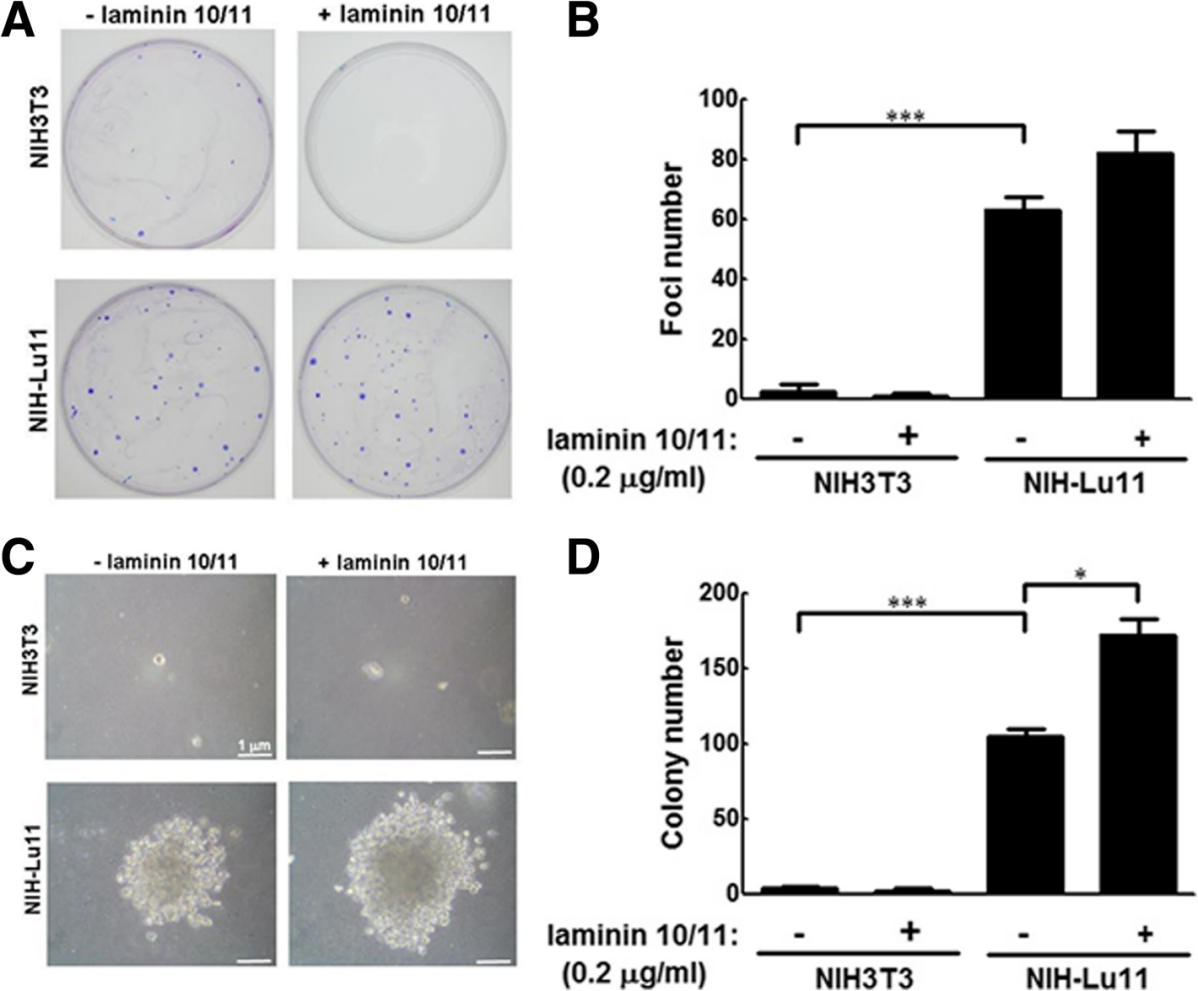
The effect of Lu/BCAM on focus and colony formation of NIH3T3 and NIH-Lu cells. **a** NIH3T3 and NIH-Lu cell lines were cultured and seeded in the presence of laminin10/11 (0.2 μg/ml) for 14 days. The cells were fixed and followed by Giemsa staining to determine focus formation. **b** The foci were counted and quantitative data are shown. This experiment was repeated three times. _***_: *p* < 0.0001. **c** Cells were cultured and seeded in 0.33% agar-containing medium with or without laminin10/11 treatment (0.2 μg/ml). The colonies were shown. **d** The size and number of colonies were measured at day 14. Colonies with a diameter greater than 3 μm were counted as positive. *: *p* < 0.05

To clarify the role of Lu/BCAM in tumor biology, the stable cell lines overexpressing Lu/BCAM transgene (pcDNA3.1-Lu) derived from NIH3T3 fibroblasts, designated as NIH-Lu10 and NIH-Lu11, were established (Additional file 1: Fig. S3). We selected NIH-Lu11 cells for further experiments because of its high expression of Lu/BCAM. Focus and anchorage-independent colony formation are two of assays to demonstrate tumorigenicity of cancer cells. We showed that foci formation of NIH-Lu11 cells in the culture plate was significantly increased compared with parental NIH3T3 cells (*p* < 0.001), and the ligand-laminin10/11 further increased foci number (Fig. 2a and b). Furthermore, we evaluated the colony-forming capability of Lu/BCAM in the presence or absence of laminin10/11 in NIH-Lu11 stable cell line. The result showed a remarkable increase of colony formation (diameter greater than 3 μm) of NIH-Lu11 cells compared to NIH3T3 cells in soft agar assay (*p* < 0.001), with a further increase of colony number in the presence of laminin10/11 (*p* < 0.05) (Fig. 2c and d). Lu/BCAM did not affect cell growth, irrespective of laminin10/11 (Additional file 1: Fig. S4). Altogether, increase of Lu/BCAM expression induces focus and colony formation via a cell growth-independent mechanism, which can be further enhanced by laminin.

### The effect of L/BCAM and its ligand laminin 10/11 on adhesion and migration of NIH-Lu11 cells

**Fig. 3:**
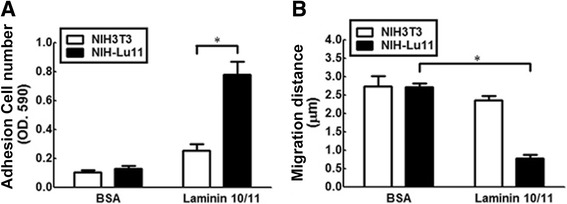
The effect of Lu/BCAM on cell adhesion and migration of NIH3T3 and NIH-Lu cells. **a** Cells were plated on the 96-well plates, which were pre-coated with laminin10/11 (40 μg/ml) or BSA (40 μg/ml). The adhesion capability of NIH-Lu 11 cells was analyzed and compared with NIH3T3 cells. **b** The cells (3 × 10^6^) were plated on a 3.5 mm cell culture dish and grown for 24 h. When cell monolayer was formed, a wound was created by carving a line using yellow tip. The wound healing process was recorded at an interval of 20 min for 24 h. All of the experiments were repeated three times.*p < 0.01

Immunoglobulin superfamily proteins, such as MUC18,  function as adhesion molecules and their expression correlates with cancer metastasis and poor prognosis [26]. It has been reported that Lu/BCAM modulates cell-matrix adhesion and/or migration in epithelial cell carcinogenesis [12]. Here, we clarified whether Lu/BCAM affects cell adhesion and migration. Our data showed that cell adhesion was significantly enhanced in NIH-Lu11 stable cell line compared with NIH3T3 cells in the presence of laminin10/11 (Fig. 3a). However, cell migration was decreased when Lu/BCAM was overexpressed in the presence of laminin10/11 (Fig. 3b). No difference of adhesion or cell migration was detected between NIH-Lu11 and NIH3T3 cells when plates were pre-coated with BSA (Fig. 3a and b). Taken together, Lu/BCAM overexpression in the presence of its ligand-laminin10/11 induced cell adhesion and consequently suppressed cell migration.

### The roles of RhoA and Rac-1 in laminin/Lu/BCAM- related cell adhesion

**Fig. 4:**
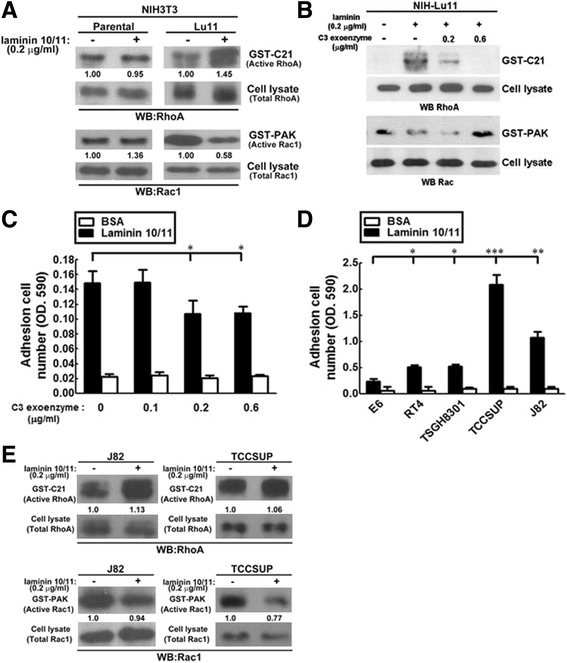
The effect of Lu/BCAM together with laminin on the activities of RhoA and Rac1 for cell adhesion of NIH-Lu 11 and bladder cancer cells. **a** Activities of RhoA (*upper panel*) and Rac1 (*lower panel*) in NIH3T3 and NIH-Lu 11 cells in the presence of laminin were evaluated by GST pull-down assay. **b** The effect of Lu/BCAM with or without laminin on the activities of RhoA and Rac1 in the presence of the RhoA inhibitor- C3 exoenzyme. Cells were treated with C3 for 1 h and whole cell lysate was prepared for pull-down by GST-PAK or -C21 protein, followed by Western blotting for activities of RhoA and Rac-1. **c** Cells were pretreated with C3 of various dosages and plated on 96-well plates, which were pre-coated with laminin 10/11 or BSA. The adhesion ability of NIH-Lu11 cells was then measured and quantitated. These experiments were repeated three times. *: *p* < 0.05. **d** The adhesion of five uroepithelial cell lines was further evaluated by cell adhesion assay in the presence of laminin or BSA.*: *p* < 0.05; **: *p* < 0.01; ***: *p* < 0.001. **e** Activities of RhoA and Rac1 in TCCSUP and J82 cell lines in the presence of laminin were determined by GST-pull down assay followed by Western blotting to illustrate active form or total amount of RhoA and Rac1. The number indicated under each band represents fold difference of active form of RhoA and Rac1 with laminin compared to RhoA and Rac1 without laminin (set as 1). In addition, values were normalized with total RhoA and Rac1 in the cell lysate first

Small GTP-binding proteins RhoA and Rac1 are known to regulate cytoskeleton rearrangement which is involved in cell-cell or cell-matrix adhesion [27, 28]. These two proteins are induced by a wide range of extracellular factors [29, 30]. To clarify their possible role in Lu/BCAM-overexpression stable cells, we first determined the activities of RhoA and Rac1 by GST-C21 (containing RhoA effector) and GST-PAK (containing Rac1 effector) pull-down analysis, respectively. Activity of RhoA (GST-C21) was slightly increased in Lu/BCAM overexpression NIH-Lu11 cells in the presence of laminin10/11 compared with parental NIH3T3 cells (Fig. 4a, upper panel). In contrast, activity of Rac1 (GST-PAK) was also slightly decreased under the same conditions compared with parental NIH3T3 cells (Fig. 4a, lower panel). This inverse relationship between RhoA and Rac-1 was confirmed using RhoA inhibitor C3 toxin, which dose-dependently suppressed RhoA activity and accordingly increased Rac-1 activity (Fig. 4b).

Furthermore, cell adhesion in the presence of laminin 10/11, but not BSA, was significantly suppressed by RhoA inhibitor C3 at concentrations of 0.2 and 0.6 μg/ml, respectively (Fig. 4c). We also examined cell adhesion using five human bladder cancer cell lines in the presence of laminin10/11 or BSA (Fig. 4d). The strength of cell adhesion was positively correlated with expression levels of Lu/BCAM after laminin10/11 treatment (Fig. 4d vs. Fig. 1B). We further selected two bladder cancer lines J82 and TCCSUP showing high expression of Lu/BCAM to measure the activity of Rho and Rac1 in the presence of laminin10/11. We demonstrated consistent results as above. Laminin10/11 together with Lu/BCAM slightly increased RhoA activity and reduced Rac1 activity in both J82 and TCCSUP cell lines (Fig. 4e). In summary, our findings imply that laminin10/11-Lu/BCAM mediated increase of cell adhesion is partially through RhoA and Rac1-related signaling pathways, and this event can be demonstrated in various cell lines, including human bladder cancer cells.

### The effect of Lu/BCAM on F-actin rearrangement in the presence of laminin

**Fig. 5:**
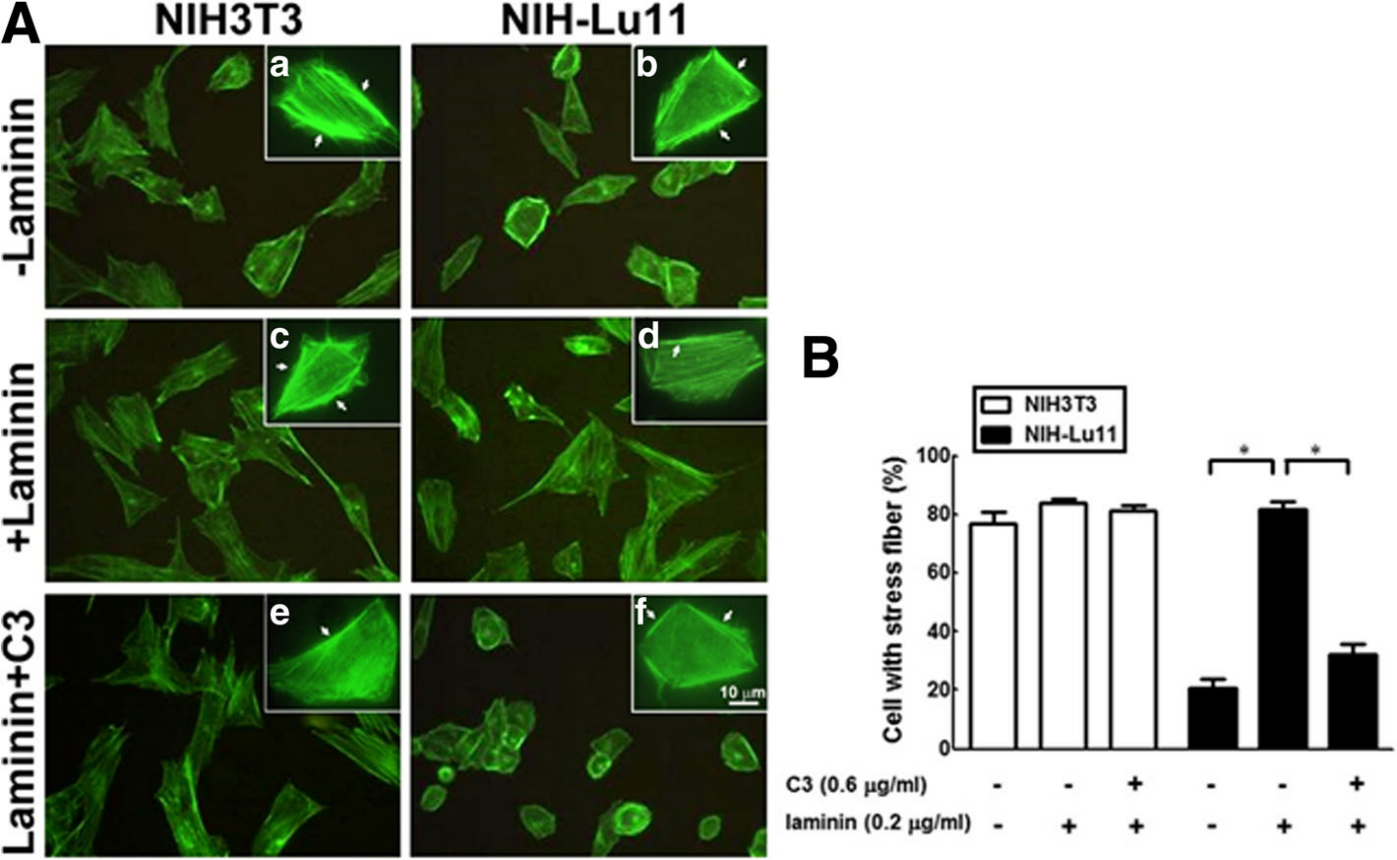
The effect of Lu/BCAM overexpression in the presence of laminin on F-actin arrangement. (**A**) Cells were treated with or without laminin and stained with Alexa Fluor™ 488-conjugated phalloidin. The **a**–**f** inserted pictures were enlargement of the image. *Arrows* indicate the aggregation of F-actin. (**B**) The quantitative data from panel (**A**) showed the numbers of cells with increased scattering distribution of F-actin. *: *p* < 0.05

Polymerization of monomer globular actin (G-actin) into filamentous actin (F-actin) leads to formation of actin stress fibers, which can be stained by phalloidin. Actin-related cytoskeleton and adhesion complex are regulated by small G protein-related signalling pathway in response to ligand stimulation to its membrane receptor [31, 32]. Here, we observed the stress fiber formation using Alexa Fluor 488-conjugated phalloidin in Lu/BCAM overexpression stable cell line after ligand laminin10/11 treatment. Our data showed that distribution of stress fibers was located around cell membrane of Lu//BCAM overexpressed cells compared with parental NIH3T3 cells (Fig. 5A–a vs. 5A–b). Stress fiber formation was significantly increased in NIH-Lu11 cells in the presence of laminin10/11 compared with those cells without laminin10/11 (Fig. 5A-d vs. 5A–b). The increased stress fiber formation in NIH-Lu11 cells mediated by laminin10/11 was suppressed by RhoA inhibitor C3 toxin (Fig. 5A-f vs. 5A–d). Quantification of stress fibers in Fig. 5A images shown in Fig. 5B indicates that RhoA participles in the laminin10/11-Lu/BCAM mediated scattering distribution of the stress fibers.

### The role of Erk phosphorylation in laminin/Lu-related RhoA/Rac1 activity, F-actin rearrangement and adhesion of NIH-Lu 11 cells

**Fig. 6:**
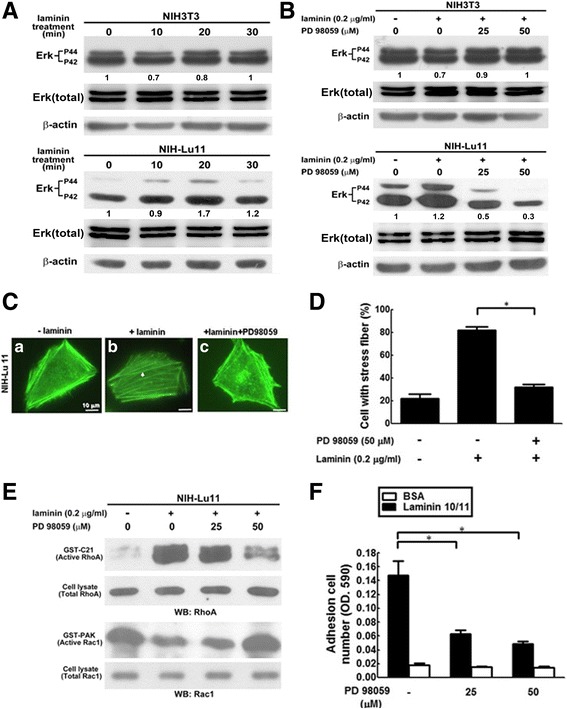
The role of Erk phosphorylation in Lu/BCAM-laminin-related Rac/Rho activity, F-actin arrangement and adhesion of NIH-Lu 11 cells. (**A**) Phosphorylation of Erk in NIH-Lu11 cells after laminin treatment for different time periods were investigated using Western blotting. (**B**) The phosphorylation of Erk was evaluated after pretreatment with MEK1/2 inhibitor PD98059 for 1 h at various dosages. (**C**) F-actin distribution in NIH-Lu11 cells with or without laminin and in the presence or absence of PD98059 was labeled with Alexa Fluor™ 488-conjugated phalloidin. **a**: Cells without any treatment; **b**: Cells treated with laminin (0.2 μg/ml); **c**: Cells treated with laminin (0.2 μg/ml) and PD98059 (50 μM) *Arrow*: polymeric F-actin. (**D**) The quantitative data of (**C**) showed those cells with scattering F-actin distribution. (**E**) NIH-Lu11 cells were pretreated with PD98059 at various dosages in the presence of laminin. Activities of RhoA and Rac-1 were evaluated by GST-C21 and GST-PAK pull-down assay, respectively, followed by Western blotting. (**F**) The adhesion ability of NIH-Lu11 cells was evaluated by pretreating cells with PD98059 for 1 h followed by plating cells on 96-well plates pre-coated with laminin 10/11 or BSA. The adhesion ability of NIH-Lu11 cells was then measured. This experiment was repeated three times. *: *p* < 0.05

**Fig. 7:**
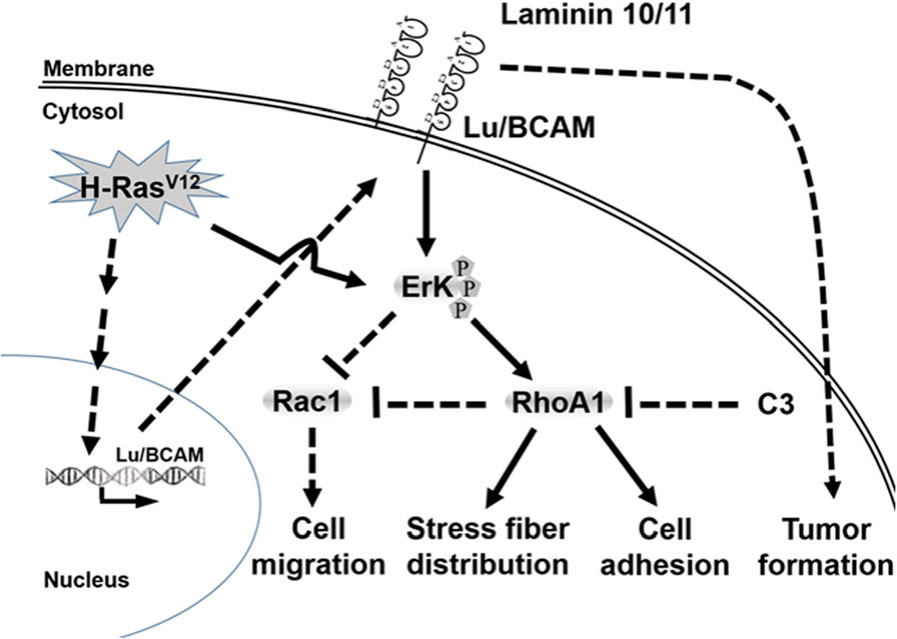
A hypothetical model of Lu/BCAM-laminin-mediated F-actin rearrangement, cell adhesion, migration and tumor formation during bladder tumorigenesis. Under Lu/BCAM overexpression conditions in the presence of laminin, the Erk/MAPK signaling pathway is activated, which is responsible for activation of RhoA and suppression of Rac-1. Subsequently, F-actin rearrangement and cell adhesion are induced, and cell migration was suppressed

The motility and metastasis of tumor cells are controlled by RhoA and Rac1 [30], and activation of ERK participates in stress fiber formation through inhibition of RhoA [33]. The signalling pathways coordinated with RhoA and Rac1 activities play pivotal roles in tumor metastasis. To clarify the role of Erk-related signalling pathway in laminin10/11-Lu/BCAM mediated cell adhesion, NIH-Lu11 cells were used. We showed an increase of Erk phosphorylation (p44 and p42) at 10 and 20 min after laminin10/11 treatment (Fig. 6A, lower panel). The MEK1/2 inhibitor PD98059 suppressed laminin10/11-induced p44 and p42 Erk phosphorylation dose-dependently (Fig. 6B, lower panel). Stress fiber formation and cell adhesion were also inhibited by PD98059 (Fig. 6C, D and F). Likewise, activity of RhoA was increased and Rac1 was decreased in laminin10/11 treated NIH-Lu11 cells, which can be further reversed by PD98059 treatment (Fig. 6E). It has been reported that both c-Jun kinase (JNK) and p38 MAPK signalling molecules are involved in cell adhesion [34]; however, laminin10/11 treatment of Lu/BCAM overexpressed cells showed no effect on these two molecule-related signaling pathways (Additional file 1: Fig. S5). Taken together, interaction of laminin and Lu/BCAM activates RhoA and suppresses Rac1 through Erk-related signaling pathway, which in turn promotes cell adhesion and inhibits cell migration through affecting stress fiber formation (Fig. 7).

## Discussion

Regulation of tumor cell adhesion and migration affects tumorigenicity during cancer development. Active mutants of *Ras* genes participate in the carcinogenesis of human cancers, including cell proliferation, mobility and tumor progression [35]. Here, we revealed that mutant form H-*ras* ^*V12*^ could transcriptionally up-regulate Lu/BCAM expression. Activation of Lu/BCAM overexpressed cells by its ligand laminin10/11 further enhances colony formation, anchorage-independent cell growth, adhesion accompanied by decreased cell migration through Erk signaling pathway, elevation of RhoA and suppression of Rac1 activity. The hypothetical model of signaling pathway for laminin stimulation of Lu/BCAM mediated cell adhesion during human bladder tumorigenesis was shown in Fig. 7.

Activation of Ras-mediated downstream signaling pathway can up-regulate protein expression of Lu/BCAM through transcriptional activation of its promoter. The distribution of Lu/BCAM protein around plasma membrane of bladder cancer cells (Fig. 1E-d) and at basal compartment of bladder cancer tissue is consistent with finding of Lu/BCAM in epithelial skin tumor [10]. This “inside-out” signaling has been reported in breast cancer cells by which Ras up-regulates vimentin and then transcriptionally activates receptor tyrosine kinase Axl to induce tumor migration and invasion [36]. Therefore, this cellular regulation path shows the great potential in the development of novel cancer therapies [37]. We further disclosed that overexpression of Lu/BCAM suppressed endogenous Ras expression in a dosage dependent manner in T24 bladder cancer cells, indicating the existence of a feedback regulation (Additional file 1: Fig. S6). Nevertheless, biological significance and the underlying mechanism by which Lu/BCAM suppresses Ras expression deserves further exploration.

Membrane proteins, including MUC18, CA199 and CD47, could enhance cell adhesion [26, 38–40], therefore show highly potential as tumor progression markers. The Lu/BCAM gene encodes two isoform proteins, which show only 40 amino acid difference at the c-terminus of protein, although both of them are single-pass transmembrane glycosylated immunoglobulin protein. We demonstrated that laminin10/11 stimulated Lu/BCAM increases cell adhesion, but suppresses cell migration. Activation of Lu/BCAM was controlled by the c-terminal 40 amino acids containing a proline-rich motif and binds to Src homology 3 domains, including the phosphorylation motifs involved in intracellular signal transduction [41]. Thus, multiple signaling pathway-related molecules, such as protein kinase A, cyclic AMP and Rap1, are involved in diverse functions of Lu/BCAM [42–44]. Previously studies have reported the importance of Lu/BCAM in the progression of several types of cancer [9, 10, 38], although the underlying mechanism remains unclear. In this study we discovered the Erk-related signaling pathway of Lu/BCAM in the tumorigenesis of human bladder. Our findings lead to the hypothesis that Lu/BCAM, in the presence of laminin, can undergo ErK-RhoA/Rac1 signaling pathway to promote F-actin rearrangement and increase tumor cell adhesion and decrease cell migration.

Lu/BCAM has been defined as adhesion receptor of laminin α5-chain [45], such as Laminin-10, −11 and −15 [46] which are responsible for cell adhesion in normal and sickle red blood cells [20, 22, 46], human erythroleukemia cells [19, 46] and murine fibroblasts [46]. Our data are consistent with Vainionpaa et al.*’*s report [7] showing that activation of Lu/BCAM by laminin10/11 can increase foci formation, colony formation, cell adhesion, scatter distribution of F-actin and tumor formation. More importantly, activation of Lu/BCAM in the presence of its ligand-laminin10/11 significantly enhances tumorigenicity, implying that ECM-tumor cells interaction might deteriorate cancer progression (Additional file 1: Fig. S7). In contrast, some studies reported that B-CAM expression was reduced in metastatic colon cancer cell lines [47], rat hepatoma [48] and malignant thyroid cancer [49]. The discrepancy between our studies and other reports is possibly explained by difference of tumor and tissue types examined. Although NIH3T3 is a mouse fibroblast cell line, the biological effects observed in NIH-Lu11 stable cells may not be faithfully recapitulated in human bladder cancer cells. Nonetheless, the phenomena observed in NIH-Lu11 cells were also demonstrated in J82 and TCCSUP human bladder cancer cell lines. It suggests that activation of Lu/BCAM and the downstream Erk signalling pathway by laminin to connect small GTPase activity (increase activity of RhoA and decrease activity of Rac1) may be a general event in tumorigenesis.

RhoA and Rac1 are the most important modulators to regulate arrangement of microfilaments and cell motility in a cooperative manner [33]. We showed that laminin stimulates Lu/BCAM to increase the adhesion of NIH-Lu and bladder cancer cells through RhoA/Rac1 signalling pathway. The positive effect of RhoA on cell adhesion, as well as its inhibitory effect on Rac activity, was further confirmed by a pharmaceutical inhibitor of Rho– C3 toxin. We showed that activity of Rac1 is inversely correlated with RhoA. It has been reported that Rho participates in the formation of actin stress fibers and focal adhesion [29, 50]. Active RhoA or RhoC can recruit a Rho kinase (ROCK), which promotes the stress fiber formation [51]. Stress fiber formation induced by ROCK might be mediated by increased phosphorylation of myosin light chain [52]. However, ROCK-induced stressfiber formation is not identical to that of Rho-induced stressfiber formation, implying that different factor(s) is involved. Another effector connecting Rho to actin cytoskeleton is mDia (also known as p140mDia), because N-terminal portion of mDia interacts with GTP-bound Rho [53]. Further exploration of Rho-related signaling pathway regulated by Lu/BCAM-laminin to induce cell adhesion is needed.

Besides, interaction of tumor cells with ECM directly activates diverse intracellular signaling molecules, such as focal adhesion kinase (FAK), Erk, JNK and p38 kinase. In NIH-Lu stable cells, we found that Erk phosphorylation after laminin treatment could be effectively inhibited by MEK1/2 inhibitor, PD98059. Subsequently, treatment of NIH-Lu cells with PD98059 also disturbed the distribution of F-actin and suppressed cell adhesion. We further revealed that blockage of Erk phosphorylation by PD98059 only partially suppressed RhoA activity and increased Rac1 activity. Together, our results suggest that Lu/BCAM and laminin together may activate Erk/MAPK or another unknown pathway to activate RhoA and suppress Rac1 which in turn affect F-actin distribution and cell adhesion. However, blocking of Erk phosphorylation could not completely inhibit cell adhesion, indicating that other signaling pathway(s) may participate in regulating cell adhesion (Fig. 7). However, whether Erk phosphorylation participates in cell migration needs further clarification. During tumor progression, oncogenic factors may trigger diverse tumorigenic activities, including increased cell proliferation, migration, focus and colony formation, angiogenesis and tumor formation. However, these tumorigenic activities induced by specific oncogenic factor, such as Lu/BCAM, are not necessarily associated. Likewise, we found that retinoblastoma binding protein-7 (RbAp46) induced by Ras increases cancer cell metastasis [54], but suppresses the tumor formation [55]. It is probable that Lu/BCAM affects cancer cell proliferation and adhesion using different mechanisms, and threshold of each tumorigenic activity may determine the direction of tumor progression. Nonetheless, the mechanism underlying Lu/BCAM promotes cell adhesion with increased tumorigenicity needs further exploration.

Our study adds Lu/BCAM to the growing list of cell adhesion molecules involved in cancer progression. Because compounds for cell surface and intracellular targets are becoming increasingly available, a prospective clinical study is needed to verify the involvement of Lu/BCAM-laminin associated pathways in anti-adhesion strategies in cancer therapies. Previous studies showed that the Erk/MAPK signaling to RhoA is via transcriptional up-regulation of Fra-1 transcription factor and to Rac1 via transcriptional up-regulation of urokinase receptor. Furthermore, Gab1, a member of docking protein family, can associate directly with phosphorylated Erk 1/2 to regulate cell motility. Whether these effects involve in Lu/BCAM-laminin related signaling pathway deserves further investigation (Fig. 7). Based on our findings, we hypothesize that human Lu/BCAM may play an active role inurothelial carcinogenesis. Interaction of laminin and Lu/BCAM activates the MEK/Erk signalling pathway to coordinate regulation of RhoA and Rac-1 in controlling microfilament distribution, cell adhesion and mobility. To develop novel human urothelial cancer therapy, further clarification of the significance and mechanism of Lu/BCAM-laminin in tumorigenesis is warranted.

## Conclusions

Over-expression of Lu/BCAM in the mouse fibroblast NIH3T3 cells increased foci number, colony formation and cell adhesion accompanied by increased F-actin stress fiber assembly and decreased cell migration compared to parental NIH3T3 cells. Lu/BCAM functions as an important adhesion molecule in response to ECM, such as laminin. Activation of Lu/BCAM by ligand laminin-10/11 induces cell adhesion which was resulted from rearrangement of stress fiber assembly through increased phosphorylation of Erk accompanied by an increase of Rho and a decrease of Rac activity. Our study supports for Lu/BCAM as a potential target for suppression of cancer malignancy.

## Additional file

Additional file 1: Table S1. C-DNA Microarray screening of H-rasV12 up-regulated genes in the bladder cancer cells E6RC compared to parental E6 cells. **Figure S1.** Lu/BCAM expression in human breast cancer cell lines and knockdown experiments. **Figure S2.** Correlation between Lu expression level and disease-specific survival. **Figure S3.** The construct of pCDNA3.1-Lu and expression of exogenic Lu in the NIH3T3 stable cell lines. **Figure S4.** The effect of Lu expression on cell proliferation in the presence or absence of laminin. **Figure S5.** The effect of Lu and laminin together on the expression levels of JNK, JNK-P, p38 and p38-P in NIH-Lu11 cells. **Figure S6.** The effect of exogenic Lu expression on Ras protein expression in T24 cells. **Figure S7.** Tumor formation of NIH-Lu and NIH3T3 cells with or without laminin in a xenograft NOD/SCID mouse model. (DOCM 1085 kb)

## Abbreviations

BCAM: Basal cell adhesion molecule
BSA: Bovine Serum Albumin
CAMs: Cell adhesion molecules
ECM: Extracellular matrix
GST: Glutathione S transferase
IFA: Immunofluorescence
Ig-CAMs: Immunoglobulin-like CAMs
IHC: Immunohistochemistry
IPTG: Isopropyl β-D-1-thiogalactopyranoside
Lu: Lutheran
MMLV: Moloney murine leukemia virus
PPIA: Peptidyl prolylisomerase A
RT-PCR: Reverse transcription-polymerase chain reaction

## References

[1] McAllister SS, Weinberg RA (2014). The tumour-induced systemic environment as a critical regulator of cancer progression and metastasis. Nat Cell Biol.

[2] Yao Y, Gu X, Liu H, Wu G, Yuan D, Yang X, Song Y (2014). Metadherin regulates proliferation and metastasis via actin cytoskeletal remodelling in non-small cell lung cancer. Br J Cancer.

[3] Akimov SS, Belkin AM (2003). Opposing roles of Ras/Raf oncogenes and the MEK1/ERK signaling module in regulation of expression and adhesive function of surface transglutaminase. J Biol Chem.

[4] Danen EH, Sonneveld P, Sonnenberg A, Yamada KM (2000). Dual stimulation of Ras/mitogen-activated protein kinase and RhoA by cell adhesion to fibronectin supports growth factor-stimulated cell cycle progression. J Cell Biol.

[5] Koontongkaew S (2013). The tumor microenvironment contribution to development, growth, invasion and metastasis of head and neck squamous cell carcinomas. J Cancer.

[6] Li DM, Feng YM (2011). Signaling mechanism of cell adhesion molecules in breast cancer metastasis: potential therapeutic targets. Breast Cancer Res Treat.

[7] Vainionpaa N, Kikkawa Y, Lounatmaa K, Miner JH, Rousselle P, Virtanen I (2006). Laminin-10 and Lutheran blood group glycoproteins in adhesion of human endothelial cells. American journal of physiology. Cell physiology.

[8] Kikkawa Y, Sanzen N, Sekiguchi K (1998). Isolation and characterization of laminin-10/11 secreted by human lung carcinoma cells. Laminin-10/11 mediates cell adhesion through integrin alpha3 beta1. J Biol Chem.

[9] Kikkawa Y, Sudo R, Kon J, Mizuguchi T, Nomizu M, Hirata K, Mitaka T (2008). Laminin alpha 5 mediates ectopic adhesion of hepatocellular carcinoma through integrins and/or Lutheran/basal cell adhesion molecule. Exp Cell Res.

[10] Schon M, Klein CE, Hogenkamp V, Kaufmann R, Wienrich BG, Schon MP (2000). Basal-cell adhesion molecule (B-CAM) is induced in epithelial skin tumors and inflammatory epidermis, and is expressed at cell-cell and cell-substrate contact sites. The Journal of investigative dermatology.

[11] Bernemann TM, Podda M, Wolter M, Boehncke WH (2000). Expression of the basal cell adhesion molecule (B-CAM) in normal and diseased human skin. J Cutan Pathol.

[12] Maatta M, Butzow R, Luostarinen J, Petajaniemi N, Pihlajaniemi T, Salo S, Miyazaki K, Autio-Harmainen H, Virtanen I (2005). Differential expression of laminin isoforms in ovarian epithelial carcinomas suggesting different origin and providing tools for differential diagnosis. J Histochem Cytochem.

[13] Yu KH, Barry CG, Austin D, Busch CM, Sangar V, Rustgi AK, Blair IA (2009). Stable isotope dilution multidimensional liquid chromatography-tandem mass spectrometry for pancreatic cancer serum biomarker discovery. J Proteome Res.

[14] Mattingly RR, Sorisky A, Brann MR, Macara IG (1994). Muscarinic receptors transform NIH 3T3 cells through a Ras-dependent signalling pathway inhibited by the Ras-GTPase-activating protein SH3 domain. Mol Cell Biol.

[15] Cheng HL, Trink B, Tzai TS, Liu HS, Chan SH, Ho CL, Sidransky D, Chow NH (2002). Overexpression of c-met as a prognostic indicator for transitional cell carcinoma of the urinary bladder: a comparison with p53 nuclear accumulation. Journal of clinical oncology : official journal of the American Society of Clinical Oncology.

[16] Su SJ, Yeh TM, Lei HY, Chow NH (2000). The potential of soybean foods as a chemoprevention approach for human urinary tract cancer. Clinical cancer research : an official journal of the American Association for Cancer Research.

[17] Kasid A, Lippman ME, Papageorge AG, Lowy DR, Gelmann EP (1985). Transfection of v-rasH DNA into MCF-7 human breast cancer cells bypasses dependence on estrogen for tumorigenicity. Science.

[18] Sommers CL, Papageorge A, Wilding G, Gelmann EP (1990). Growth properties and tumorigenesis of MCF-7 cells transfected with isogenic mutants of rasH. Cancer Res.

[19] Parsons SF, Lee G, Spring FA, Willig TN, Peters LL, Gimm JA, Tanner MJ, Mohandas N, Anstee DJ, Chasis JA (2001). Lutheran blood group glycoprotein and its newly characterized mouse homologue specifically bind alpha5 chain-containing human laminin with high affinity. Blood.

[20] Zen Q, Cottman M, Truskey G, Fraser R, Telen MJ (1999). Critical factors in basal cell adhesion molecule/lutheran-mediated adhesion to laminin. J Biol Chem.

[21] Yeh HH, Lai WW, Chen HH, Liu HS, Su WC (2006). Autocrine IL-6-induced Stat3 activation contributes to the pathogenesis of lung adenocarcinoma and malignant pleural effusion. Oncogene.

[22] Udani M, Zen Q, Cottman M, Leonard N, Jefferson S, Daymont C, Truskey G, Telen MJ (1998). Basal cell adhesion molecule/lutheran protein. The receptor critical for sickle cell adhesion to laminin. J Clin Invest.

[23] Hsu PY, Liu HS, Cheng HL, Tzai TS, Guo HR, Ho CL, Chow NH (2006). Collaboration of RON and epidermal growth factor receptor in human bladder carcinogenesis. J Urol.

[24] Liu HS, Scrable H, Villaret DB, Lieberman MA, Stambrook PJ (1992). Control of ha-ras-mediated mammalian cell transformation by Escherichia Coli regulatory elements. Cancer Res.

[25] Yeh CY, Shin SM, Yeh HH, Wu TJ, Shin JW, Chang TY, Raghavaraju G, Lee CT, Chiang JH, Tseng VS, Lee YC, Shen CH, Chow NH, Liu HS (2011). Transcriptional activation of the Axl and PDGFR-alpha by c-met through a ras- and Src-independent mechanism in human bladder cancer. BMC Cancer.

[26] Lehmann JM, Riethmuller G, Johnson JP (1989). MUC18, a marker of tumor progression in human melanoma, shows sequence similarity to the neural cell adhesion molecules of the immunoglobulin superfamily. Proc Natl Acad Sci U S A.

[27] Arthur WT, Noren NK, Burridge K (2002). Regulation of rho family GTPases by cell-cell and cell-matrix adhesion. Biol Res.

[28] Guillemot L, Paschoud S, Jond L, Foglia A, Citi S (2008). Paracingulin regulates the activity of Rac1 and RhoA GTPases by recruiting Tiam1 and GEF-H1 to epithelial junctions. Mol Biol Cell.

[29] Allen WE, Jones GE, Pollard JW, Ridley AJ (1997). Rho, Rac and Cdc42 regulate actin organization and cell adhesion in macrophages. J Cell Sci.

[30] Parri M, Chiarugi P (2010). Rac and rho GTPases in cancer cell motility control. Cell communication and signaling : CCS.

[31] Hall A (2005). Rho GTPases and the control of cell behaviour. Biochem Soc Trans.

[32] Burridge K, Wennerberg K (2004). Rho and Rac take center stage. Cell.

[33] Vial E, Sahai E, Marshall CJ (2003). ERK-MAPK signaling coordinately regulates activity of Rac1 and RhoA for tumor cell motility. Cancer Cell.

[34] Schmidmaier R, Baumann P (2008). ANTI-ADHESION evolves to a promising therapeutic concept in oncology. Curr Med Chem.

[35] Webb CP, Van Aelst L, Wigler MH, Vande Woude GF (1998). Signaling pathways in Ras-mediated tumorigenicity and metastasis. Proc Natl Acad Sci U S A.

[36] Vuoriluoto K, Haugen H, Kiviluoto S, Mpindi JP, Nevo J, Gjerdrum C, Tiron C, Lorens JB, Ivaska J (2011). Vimentin regulates EMT induction by slug and oncogenic H-Ras and migration by governing Axl expression in breast cancer. Oncogene.

[37] Takabe K, Paugh SW, Milstien S, Spiegel S (2008). "inside-out" signaling of sphingosine-1-phosphate: therapeutic targets. Pharmacol Rev.

[38] Campbell IG, Foulkes WD, Senger G, Trowsdale J, Garin-Chesa P, Rettig WJ (1994). Molecular cloning of the B-CAM cell surface glycoprotein of epithelial cancers: a novel member of the immunoglobulin superfamily. Cancer Res.

[39] Zhou G, Chiu D, Qin D, Niu L, Cai J, He L, Huang W, Xu K (2012). The efficacy evaluation of cryosurgery in pancreatic cancer patients with the expression of CD44v6, integrin-beta1, CA199, and CEA. Mol Biotechnol.

[40] Azcutia V, Routledge M, Williams MR, Newton G, Frazier WA, Manica A, Croce KJ, Parkos CA, Schmider AB, Turman MV, Soberman RJ, Luscinskas FW (2013). CD47 plays a critical role in T-cell recruitment by regulation of LFA-1 and VLA-4 integrin adhesive functions. Mol Biol Cell.

[41] Yu H, Chen JK, Feng S, Dalgarno DC, Brauer AW, Schreiber SL (1994). Structural basis for the binding of proline-rich peptides to SH3 domains. Cell.

[42] Gauthier E, Rahuel C, Wautier MP, El Nemer W, Gane P, Wautier JL, Cartron JP, Colin Y, Le Van Kim C (2005). Protein kinase A-dependent phosphorylation of Lutheran/basal cell adhesion molecule glycoprotein regulates cell adhesion to laminin alpha5. J Biol Chem.

[43] Hines PC, Zen Q, Burney SN, Shea DA, Ataga KI, Orringer EP, Telen MJ, Parise LV (2003). Novel epinephrine and cyclic AMP-mediated activation of BCAM/Lu-dependent sickle (SS) RBC adhesion. Blood.

[44] Murphy MM, Zayed MA, Evans A, Parker CE, Ataga KI, Telen MJ, Parise LV (2005). Role of Rap1 in promoting sickle red blood cell adhesion to laminin via BCAM/LU. Blood.

[45] Kikkawa Y, Moulson CL, Virtanen I, Miner JH (2002). Identification of the binding site for the Lutheran blood group glycoprotein on laminin alpha 5 through expression of chimeric laminin chains in vivo. J Biol Chem.

[46] El Nemer W, Gane P, Colin Y, D'Ambrosio AM, Callebaut I, Cartron JP, Van Kim CL (2001). Characterization of the laminin binding domains of the Lutheran blood group glycoprotein. J Biol Chem.

[47] Le Naour F, Andre M, Greco C, Billard M, Sordat B, Emile JF, Lanza F, Boucheix C, Rubinstein E (2006). Profiling of the tetraspanin web of human colon cancer cells. Molecular & cellular proteomics : MCP.

[48] Akiyama H, Iwahana Y, Suda M, Yoshimura A, Kogai H, Nagashima A, Ohtsuka H, Komiya Y, Tashiro F (2013). The FBI1/Akirin2 target gene, BCAM, acts as a suppressive oncogene. PLoS One.

[49] Latini FR, Bastos AU, Arnoni CP, Muniz JG, Person RM, Baleotti W, Barreto JA, Castilho L, Cerutti JM (2013). DARC (Duffy) and BCAM (Lutheran) reduced expression in thyroid cancer. Blood Cells Mol Dis.

[50] Ren XD, Kiosses WB, Schwartz MA (1999). Regulation of the small GTP-binding protein rho by cell adhesion and the cytoskeleton. EMBO J.

[51] Sahai E, Marshall CJ (2003). Differing modes of tumour cell invasion have distinct requirements for rho/ROCK signalling and extracellular proteolysis. Nat Cell Biol.

[52] Roovers K, Assoian RK (2003). Effects of rho kinase and actin stress fibers on sustained extracellular signal-regulated kinase activity and activation of G(1) phase cyclin-dependent kinases. Mol Cell Biol.

[53] Kaibuchi K, Kuroda S, Amano M (1999). Regulation of the cytoskeleton and cell adhesion by the rho family GTPases in mammalian cells. Annu Rev Biochem.

[54] Yeh HH, Tseng YF, Hsu YC, Lan SH, Wu SY, Raghavaraju G, Cheng DE, Lee YR, Chang TY, Chow NH, Hung WC, Liu HS (2015). Ras induces experimental lung metastasis through up-regulation of RbAp46 to suppress RECK promoter activity. BMC Cancer.

[55] Giri R, Yeh HH, Wu CH, Liu HS (2008). SUMO-1 overexpression increases RbAp46 protein stability and suppresses cell growth. Anticancer Res.

