# Supplementary material for: The role of Lutheran/basal cell adhesion molecule in human bladder carcinogenesis
Source: J Biomed Sci. 2017 Aug 26;24:61. doi: 10.1186/s12929-017-0360-x (PMC6389174; doi:10.1186/s12929-017-0360-x)
Supplement: Supplementary file 1 — C-DNA Microarray screening of H-rasV12 up-regulated genes in the bladder cancer cells E6RC compared to parental E6 cells. Figure S1. Lu/BCAM expression in human breast cancer cell lines and knockdown experiments. Figure S2. Correlation between Lu expression level and disease-specific survival. Figure S3. The construct of pCDNA3.1-Lu and expression of exogenic Lu in the NIH3T3 stable cell lines. Figure S4. The effect of Lu expression on cell proliferation in the presence or absence of laminin. Figure S5. The effect of Lu and laminin together on the expression levels of JNK, JNK-P, p38 and p38-P in NIH-Lu11 cells. Figure S6. The effect of exogenic Lu expression on Ras protein expression in T24 cells. Figure S7. Tumor formation of NIH-Lu and NIH3T3 cells with or without laminin in a xenograft NOD/SCID mouse model. (DOCM 1085 kb) [file 12929_2017_360_MOESM1_ESM.docm]

Additional file 1

Figure S1. Lu/BCAM expression in human breast cancer cell lines and knockdown experiments. (A) The mRNA expression of Lu in breast cancer MCF-7-H-*ras^V12^* cells in the presence of IPTG for the times as indicated was detected by real-time PCR. This experiment was repeated three times. The protein expression of Ras in MCF-7-*ras* cells was detected by Western blotting. (B) Ras and Lu protein expression was evaluated by Western blotting after transduction with lentivirus-driven Ras shRNAs (psh-Ras-1 and psh-Ras-2). PC: The HEK 293 cell was used as the positive control for Lu expression. NC: negative control; V: vector control.

Figure S2. Correlation between Lu expression level and disease-specific survival Correlation of Lu expression with disease-specific survival in urothelial carcinoma patients was determined by Kaplan-Meier survival analysis. A total of 60 bladder cancer patients were analyzed

Figure S3. The construct of pCDNA3.1-Lu and expression of exogenic Lu in the NIH3T3 stable cell lines. A plasmid pCDNA3.1-Lu harboring Lu gene was constructed. B: The expression levels of Lu gene in NIH3T3 and NIH-Lu 10-11 cell lines were assessed using Real-Time PCR. NIH-Lu 10 and NIH-Lu 11 are NIH3T3 derivates, constitutively express Lu gene.

Figure S4. The effect of Lu expression on cell proliferation in the presence or absence of laminin. The proliferation of NIH3T3 and NIH-Lu cells, with or without laminin 10/11treatment, was evaluated by BrdU incorporation assay in which bromodeoxyuridine (Sigma, USA) was used to label the cells.

Figure S5. The effect of Lu and laminin together on the expression levels of JNK, JNK-P, p38 and p38-P in NIH-Lu11 cells. The expression levels of JNK, JNK-P and p38, p38-P in NIH-Lu11 cells after laminin treatment were detected at various time intervals. The JNK and JNK-P proteins were recognized by JNK (Cell Signaling) and JNK-phosphorylation antibodies (Cell Signaling), respectively. The p38 and p38-P proteins were recognized by p38 and p38-P antibodies (Santa Cruz), respectively.

Figure S6. The effect of exogenic Lu expression on Ras protein expression in T24 cells. Bladder cancer T24 cells were transfected with plasmid pcDNA3.1-Lu at various amounts and Ras protein expression level was determined by Western blotting using anti-Ras antibody. GAPDH

was used as an internal control. CTRL: Negative control. V: Vector control.

Figure S7. Tumor formation of NIH-Lu and NIH3T3 cells with or without laminin in a xenograft NOD/SCID mouse model. NIH3T3 and NIH-Lu-11 cell lines with or without laminin treatment were injected subcutaneously into NOD/SCID mice. These mice were divided into four groups and each group contained three mice. Tumor volume was measured for 17 days.

Table S1. C-DNA Microarray screening of H-*ras*^V12^ up-regulated genes in the bladder cancer cells E6RC compared to parental E6 cells.

| **Name** | **Accession** | **UG_Link** | **Gene_Symbol** | **Function** | **Ration of**  **mean** |
| --- | --- | --- | --- | --- | --- |
| **Lutheran blood group** | **P50895** | **Hs.155048** | **LU** | **N/A** | **2.62** |
| B-cell translocation gene 1,  anti-proliferation | N70463 | Hs.77054 | BTG1 | Cell cycle | 2.59 |
| Protein kinase C, nu | AA463213 | Hs.143460 | PRKCN | Kinase | 2.59 |

Figure S1


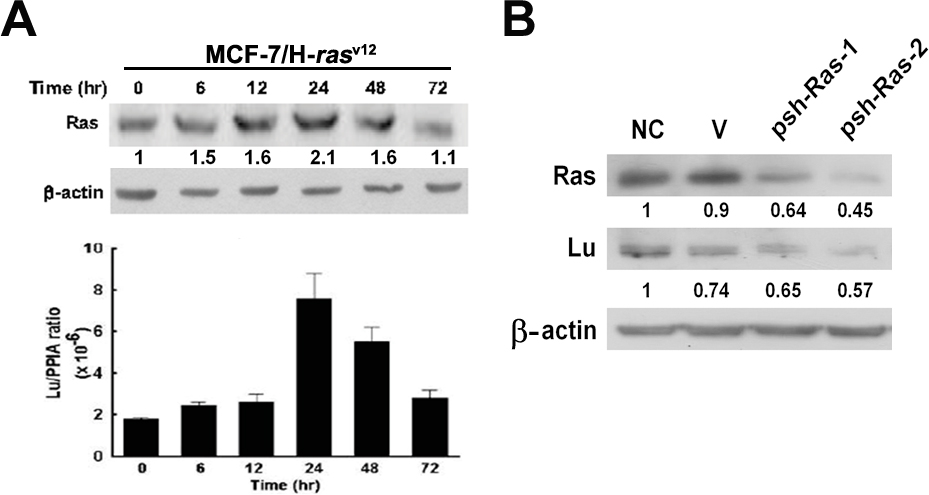


**A**


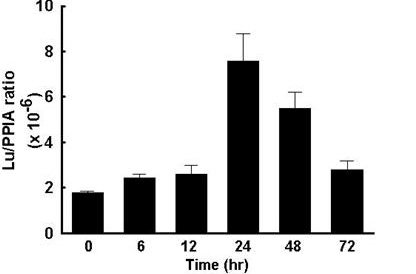


**Time (hr)**

**Ras**

**β-actin**


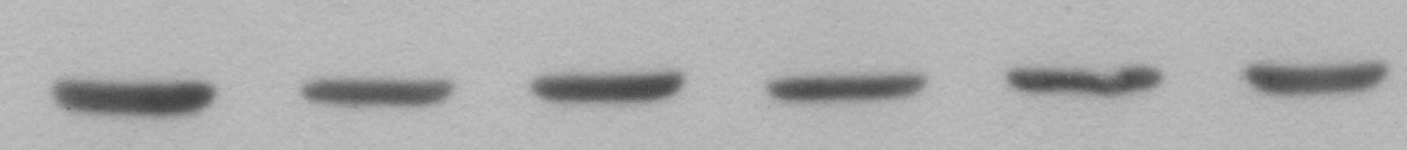


**Time (hr) 0 6 12 24**


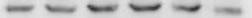


**1 1.5 1.6 2.1**

**MCF-7/H-*ras*^v12^**

Figure S2


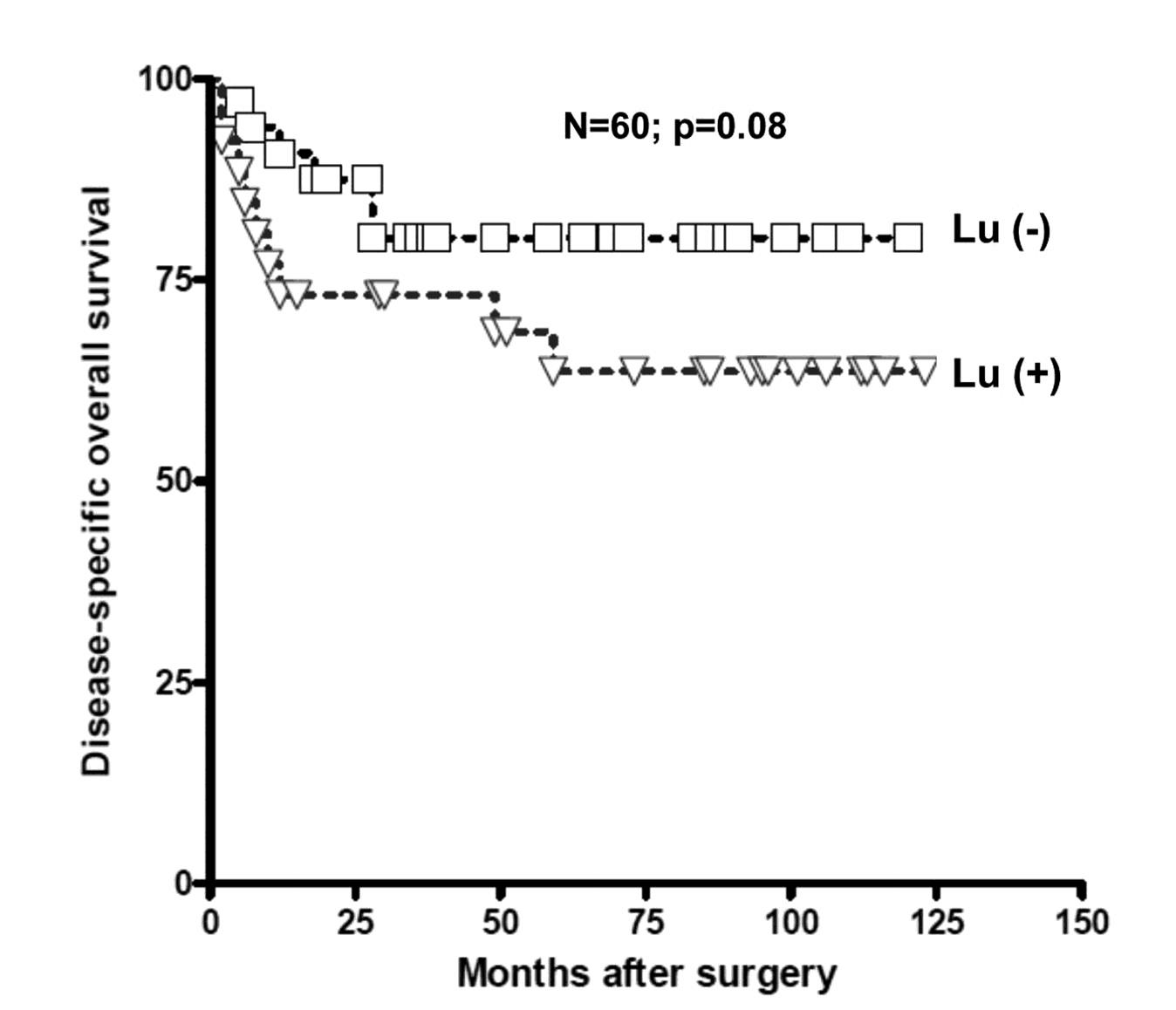


Figure S3


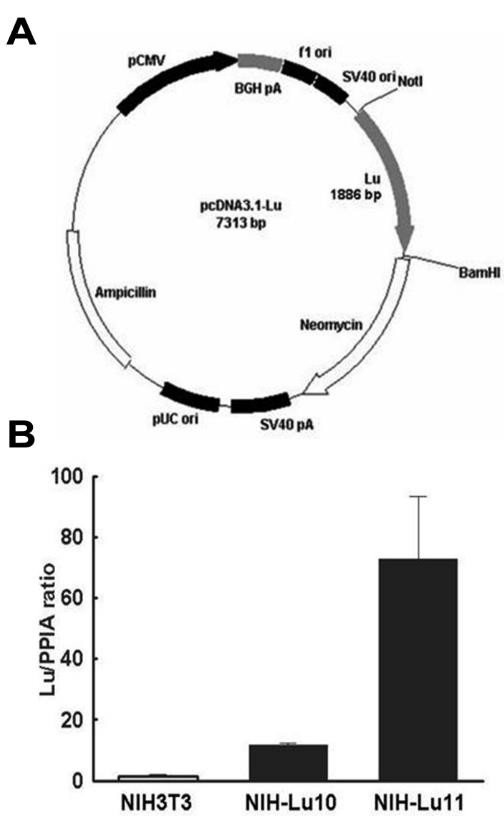


Figure S4


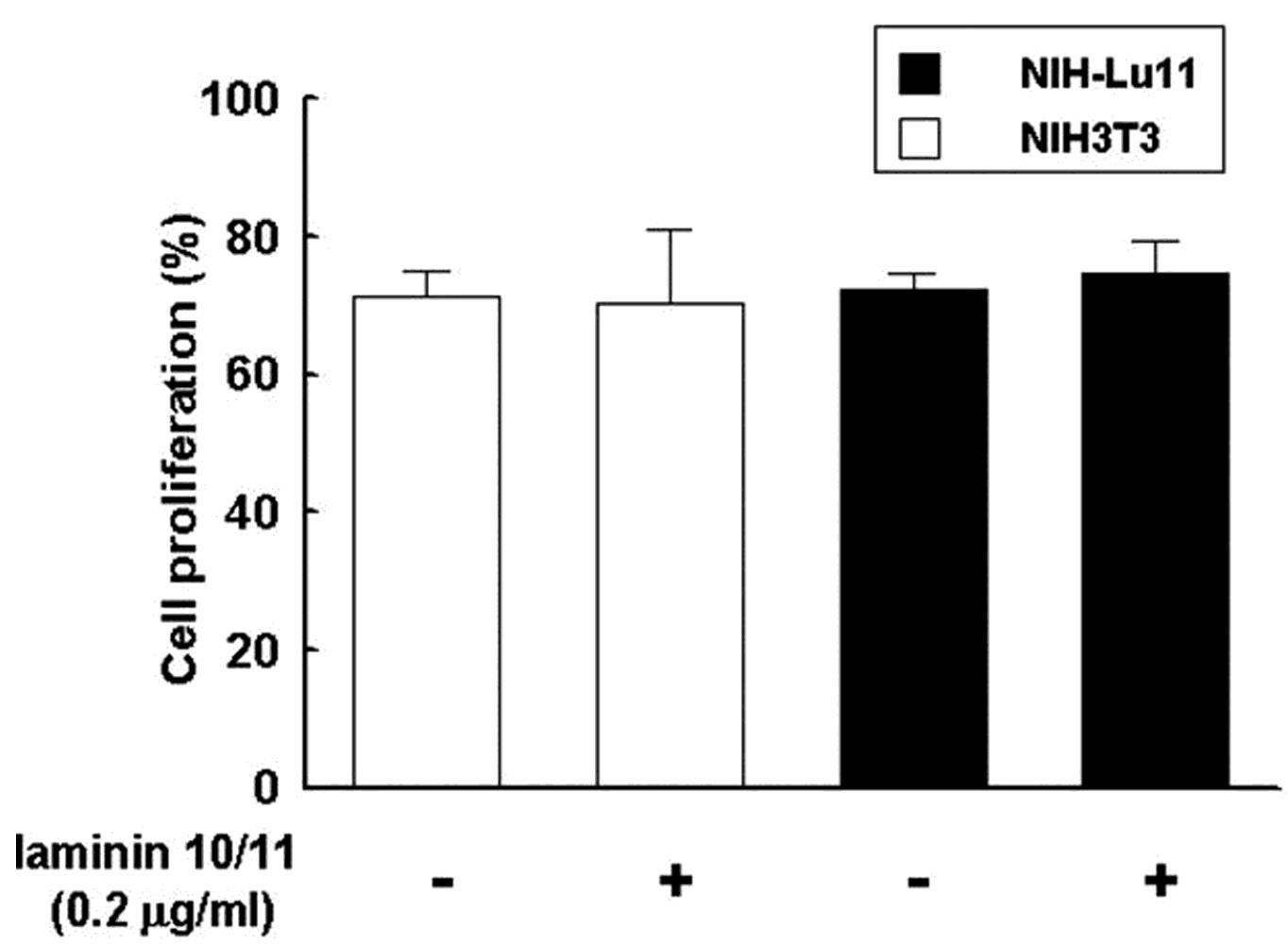


Figure S5


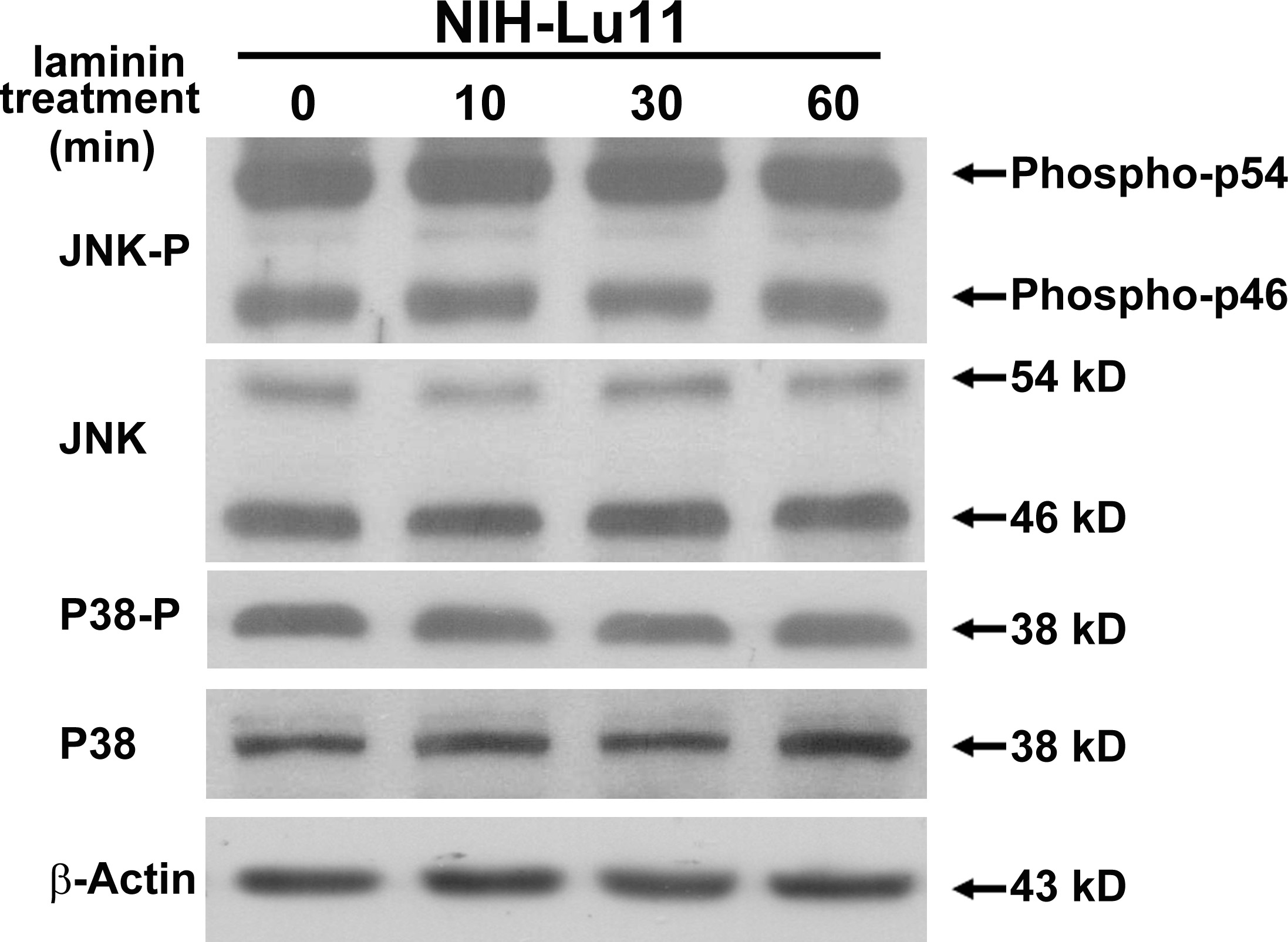


Figure S6


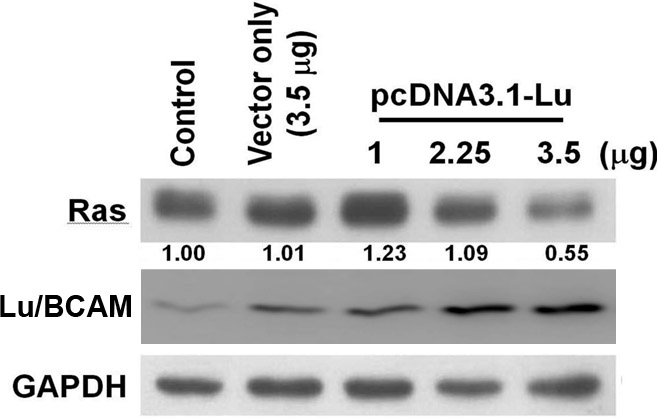


Figure S7
